# Quantitative imaging of magnetic nanoparticles in an unshielded environment using a large AC susceptibility array

**DOI:** 10.1186/s13036-022-00305-9

**Published:** 2022-10-11

**Authors:** Guilherme Soares, Leonardo Pinto, Maik Liebl, Gabriel Biasotti, Andre Prospero, Erick Stoppa, Andris Bakuzis, Oswaldo Baffa, Frank Wiekhorst, José Ricardo Arruda Miranda

**Affiliations:** 1 Physikalisch-Technische Bundesanstalt, Abbestraße 2–12, 10587 Berlin, Germany; 2 Biosciences Institute of Botucatu, São Paulo State University, Botucatu, São Paulo, 18618-689 Brazil; 3 Institute of Physics, Federal University of Goiás, Goiânia, 74690-900 Brazil; 4 Faculty of Philosophy, Sciences and Letters at Ribeirão Preto, University of São Paulo, Ribeirão Preto, Sao Paulo, 14040-900 Brazil

**Keywords:** Magnetic nanoparticles, Quantitative imaging, Alternate current biosusceptometry, Inverse problem

## Abstract

**Background:**

Non-invasive magnetic imaging techniques are necessary to assist magnetic nanoparticles in biomedical applications, mainly detecting their distribution inside the body. In Alternating Current Biosusceptometry (ACB), the magnetic nanoparticle's magnetization response under an oscillating magnetic field, which is applied through an excitation coil, is detected with a balanced detection coil system.

**Results:**

We built a Multi-Channel ACB system (MC-ACB) containing nineteen pick-up coils and obtained 2D quantitative images of magnetic nanoparticle distributions by solving an inverse problem. We reconstructed the magnetic nanoparticles spatial distributions in a field of view of 14 × 14 cm^2^ with a spatial resolution of 2.0 cm and sensitivity in the milligram scale. A correlation coefficient between quantitative reconstructed and nominal magnetic nanoparticle distributions above 0.6 was found for all measurements.

**Conclusion:**

Besides other interesting features such as sufficient large field of view dimension for mice and rat studies, portability, and the ability to assess the quantitative magnetic nanoparticles distributions in real-time, the MC-ACB system is a promising tool for quantitative imaging of magnetic nanoparticles distributions in real-time, offering an affordable setup for easy access in clinical or laboratory environments.

## Introduction

Magnetic nanoparticles (MNPs) present great versatility due to their inherent magnetic properties and reduced size, enabling many biomedical applications [1]. Reliable detection and quantification of MNPs distributions are indispensable for drug delivery or magnetic hyperthermia applications [2, 3], since this information determines if the MNPs reached the desirable site and in an appropriate amount for an adequate procedure such as hyperthermia, or a goal, as drug delivery. Therefore, several non-invasive techniques were developed and applied to perform the quantitative imaging of MNPs distributions. The highly sensitive Magnetorelaxometry (MRX) and Magnetic Particle Imaging (MPI) have been widely employed to quantify in vivo MNPs in small animals with a great spatial resolution [4, 5], and further optimizations are continually being developed [6–9]. However, present MRX and MPI devices are designed for high performance, including magnetic shielding, which makes these systems very expensive. This limits the availability of MRX and MPI systems for many research groups, making more cost-efficient techniques such as ACB attractive for MNPs imaging.

Alternatively, AC susceptibility devices were applied for quantitative imaging of MNPs detecting their AC susceptibility. Ficko and collaborators introduced three MNPs imaging methodologies based on AC susceptibility measurements, in which the authors highlighted the low-cost instrumentation of the approaches [10]. The system, denominated as susceptibility magnitude imaging (SMI), can reconstruct MNPs distribution with a spatial resolution in the order 1 cm^−1^ and real-time for a field of view (FOV) of 4.5 cm, respectively.

In this way, the ACB system applies an alternating magnetic field to magnetize the MNPs and detect their dynamic magnetic response through a pair of coils in a gradiometric configuration [11]. The single-channel ACB system provides a real-time signal acquisition and has been widely employed in studies involving magnetic microparticles and MNPs, such as in the quantification of biodistribution studies of MNPs and the detection of MNPs in vivo [12–19]. Besides, the system is already established for in vivo pre-clinical MNPs monitoring, assessing its biodistribution patterns. Previous studies successfully used the ACB system to detect and monitor the MNPs after the intravenous administration in the bloodstream, liver, kidneys, and brain in real-time [20–23]. Furthermore, a second array, known as a multi-channel ACB (MC-ACB) system with seven pairs of pick-up coils, was developed to map the biodistribution of MNPs [22]. Despite the advantages of acquisition with high temporal resolution and the potential for real-time imaging, the absence of a direct correlation between pixel intensity with MNPs mass restricts the application of the ACB system to qualitative analysis. To overcome this limitation, Biasotti and co-workers employed a scanning approach to the single-sensor ACB system by establishing a forward problem and solving the corresponding inverse problem, obtaining 2D quantitative images of an MNPs spatial distribution [24]. Though it is suitable for ex vivo biodistribution studies or in vivo quantifications limited to a specific organ, this ACB setup is limited for future real-time imaging applications due to the prolonged scanning times.

In this paper, we present the progress of improving the ACB system as a measurement tool to provide a quantitative assessment of the spatial distribution of MNPs. To this end, we established a feasible system that could be useful for real-time MNPs quantification in rodents. We increased the FOV, implemented the appropriate forward model, and solved the inverse problem for quantitative MNPs imaging. Different MNP amounts inserted in gypsum cubes were used to reconstruct the particle distribution and estimate the MC-ACB system’s spatial resolution and sensitivity.

## Material and methods

### MC-ACB system

**Fig. 1:**
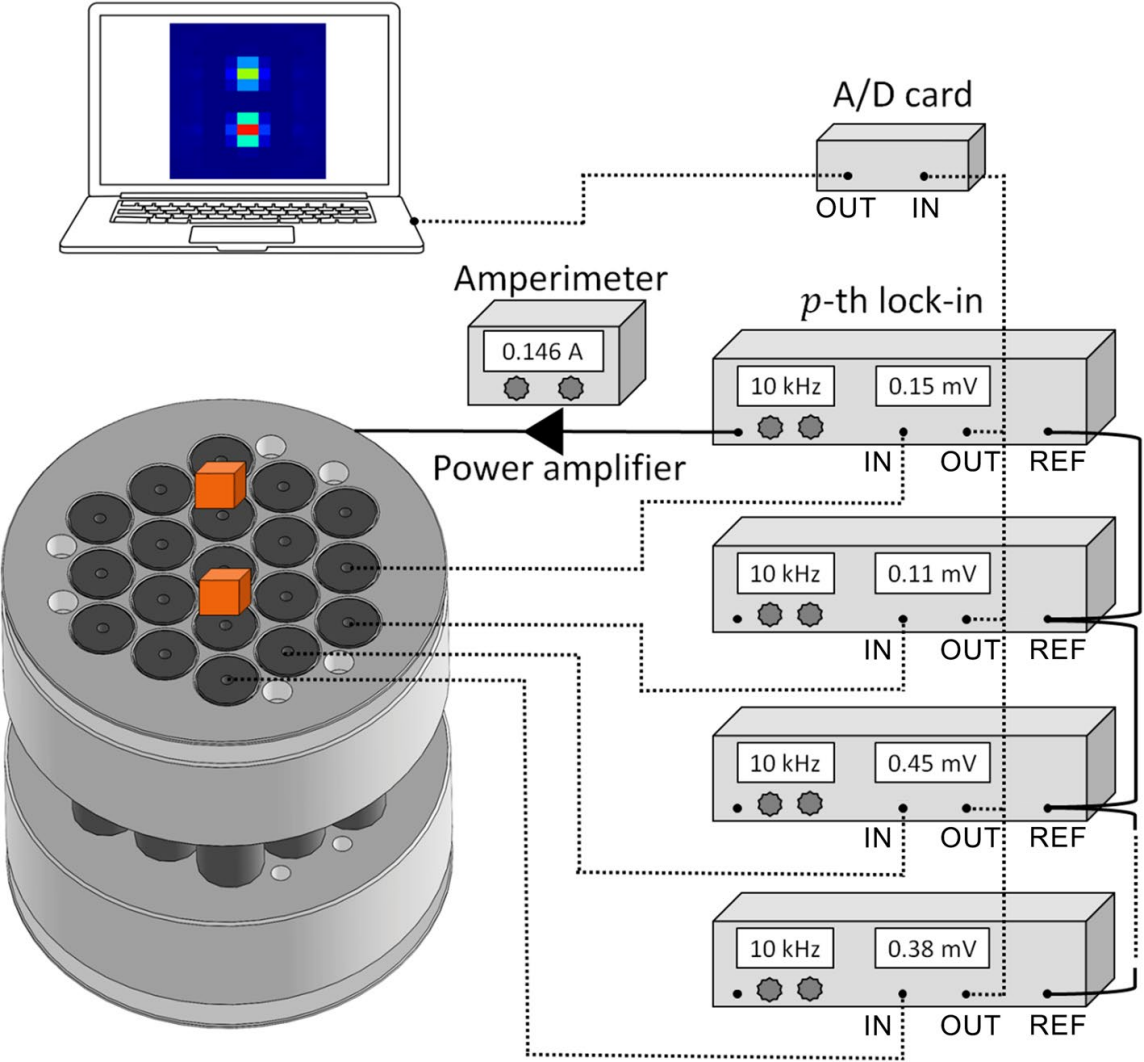
Schematic diagram of MC-ACB setup used for MNP measurements and quantitative 2D-reconstruction. A set of 19 lock-in amplifiers was used with the same reference signal that also fed the power amplifier. The current send to the excitation coils was monitored using an ammeter (Fluke-115) to keep the excitation field constant during all the signal acquisition

The MC-ACB system consists of one pair of excitation and nineteen pairs of pick-up coils. The pick-up coils are connected in a first-order gradiometric configuration to reduce the excitation field and the environmental noise, thereby increasing the system’s sensitivity. A baseline of 13.7 cm separates the coils within a pair to suppress mutual interferences. The system was developed with the excitation coils having a radius of 6.24 cm and 135 turns (AWG 20); and the pick-up coils with a radius of 0.83 cm and 1200 turns (AWG 32), arranged in a honeycomb configuration as represented in Fig. 1. The coil system was rigidly mounted in a plastic frame to avoid displacement during measurements and keep the maximum common mode signal rejection when there are no MNPs near the sensors.

Measurements were carried out using 19 lock-in amplifiers (SR830, Stanford Research Systems, USA) and an audio power amplifier (TIP 800, Ciclotron, Brazil) to apply a 2 mT magnetic field at 10 kHz in the excitation coils, which provides an inhomogeneous alternating magnetic field to magnetize the MNPs in the linear susceptibility range. The MNPs response, induced into the pick-up coils, was readout as a voltage by lock-in amplifiers. The voltage amplitude detected by the lock-in amplifier is our raw data, and it was acquired at a 20 Hz sampling rate using an A/D acquisition board (DAQPad 6015, National Instruments, USA) and LabView software.

### MC-ACB system stability and noise experiment

Stability and noise experiments were performed in the MC-ACB system at the Berlin Magnetically Shielded Room 2 (BMSR-2) of the Physikalisch-Technischen Bundesanstalt (PTB). The BMSR-2 is an eight-layered magnetically shielded room in which seven layers are Mu-metal to shield low-frequency magnetic fields while an additional layer of very high conductivity aluminum works as an eddy current barrier to attenuate high-frequency fields.

The MC-ACB was operated in its work function. The SR830 was employed to generate an electrical signal of 0.7 V at a frequency of 10 kHz and amplified by power amplifiers (− 3 dB). Both MC-ACB signals inside and outside the BSMR-2 were acquired continuously for 10 min at a 20 Hz sampling rate using the A/D board previously described. The measurement was performed using no magnetic material.

We also evaluated the system's response to different magnetic nanoparticle conjugations with varying synthesis protocols (BerlinHeart, Perimag, FluidMag – D, and manganese ferrite nanoparticles) and different sizes (FluidMag – D 50 nm, 100 nm, and 200 nm).

### Quantitative MC-ACB imaging

The mathematical approach of both the forward and inverse problems for quantitative 2D ACB imaging has been described by Biasotti et al. [24]. Briefly, considering $p$ distinct pick-up coils and a FOV composed of $v$ voxels, the induced voltage in the $p$-th pick-up coil, generated by a MNP mass (${X}_{MNP,v}$) in the $v$-th voxel, can be calculated by Faraday’s law and is given by:

1 $${V}_{p, v}=-\frac{d}{dt}\left[\frac{{\mu }_{0}}{{I}_{r}}.({{\varvec{H}}}_{{\varvec{r}},{\varvec{p}},{\varvec{v}} }\cdot {{\varvec{H}}}_{{\varvec{m}},{\varvec{p}},{\varvec{v}}}).\chi \left(\omega \right).{\rm X}_{MNP,v}\right]$$

where ${V}_{p, v}$ is the induced voltage in the *p*-th pick-up coil and the *v*-th voxel, ${\mu }_{0}$ is the magnetic permeability in a vacuum, ${I}_{r}$ is the pick-up coil reciprocal current, $\chi \left(\omega \right)$ is the frequency-dependent mass susceptibility, ${{\varvec{H}}}_{{\varvec{r}},{\varvec{p}},{\varvec{v}}}$ and ${{\varvec{H}}}_{{\varvec{m}},{\varvec{p}},{\varvec{v}}}$ are the reciprocal and the magnetizing field in the $p$-th pick-up coil and $v$-th voxel, respectively.

The separation of geometric parameters and material properties of the MNP from the MNP mass inside the $v$-th voxel in Eq. (1) can be written as [6]:

2 $${\varvec{V}}\boldsymbol{ }=\sum\nolimits_{v}{\varvec{L}}.{\rm X}_{MNP,v}={\varvec{L}}\cdot {{\varvec{X}}}_{MNP}$$

where ${\varvec{V}}$ is a vector containing the induced voltage in all pick-up coils, and ${\varvec{L}}$ is the sensitivity matrix of the system, which has the dimensions of $v \times p$ and includes the sensitivity of the $p$-th pick-up coil to a unit MNP mass in the $v$-th voxel, and ${{\varvec{X}}}_{MNP}$ is a vector containing the MNP amount in each voxel.

We solved Eq. (2) by applying the Moore–Penrose pseudo-inverse matrix (${{\varvec{L}}}^{+}={({{\varvec{L}}}^{{\varvec{T}}}{\varvec{L}}) }^{-1}{{\varvec{L}}}^{{\varvec{T}}}$), which is minimum norm estimation obtained by truncated singular value decomposition (TSVD). This approach has been applied before in quantitative MNP imaging [25, 26] and is given by:

3 $${{\varvec{X}}}_{MNP,est}= {{\varvec{L}}}^{+}.{\varvec{V}}$$

where ${{\varvec{X}}}_{MNP,est}$ is the estimated amount of MNP in each voxel.

### MNP phantoms

We employed Perimag MNPs (product code 102–00-132, Micromod Partikeltechnologie GmbH, Rostock, Germany). The MNP stock suspension has an iron concentration c(Fe) = 9.5 mg/ml, with the MNPs exhibiting a saturation magnetization M_S_ of about 90 Am^2^/kg iron (H > 800 kA/m) and a hydrodynamic diameter d_hyd_ of about 130 nm. For our measurements, the MNPs were immobilized in gypsum at different concentrations to produce 10 cubic phantoms with dimensions of *1.2* × *1.2* × *1.2* cm^3^ and an iron mass ranging from 0.05 to 4.8 mg [27].

### MC-ACB measurements

Firstly, we established the forward model to discretize our FOV in $v$ voxels, and then we determined the sensitive matrix ***L***, which was measured as the point spread function (PSF) at each possible voxel position in the FOV.

We performed measurements using the reference gypsum cube with a Fe content of 1.04 mg, positioned at a vertical distance of 0.3 cm from the pick-up coils’ surface. As the A/D board acquires signals at a rate of 20 Hz, we previously determined that the acquisition signal with immobile material would be given by the average of the signals acquired over 10 s.

The cube was moved by a 2D CNC (Computed Numeric Control) in a 14 × 14 cm^2^ grid at a step of 1.0 cm. Therefore, the selected FOV is centered according to the central detector coil with a volume of $14 x 14 x1.2 c{m}^{3}$, distributed in 196 *voxels* of $1 x 1 x 1.2 c{m}^{3}$. that was utilized for all reconstructions.

We assessed how the system would reconstruct separated cubes at a certain distance to estimate the system's sensitivity and spatial resolution.

We positioned two gypsum cubes with a total MNP content of 4.80 and 3.06 mg along the FOV’s central line, distanced at 3, 2, and 1 cm. The vertical distance between both cubes and the pick-up coils’ surface was 0.3 cm. We determined the spatial resolution as the minimum distance the system can resolve two cubes (Fig. 6).

Furthermore, due to the recent application of the MC-AC system in the evaluation of hepatic retention and cardiac perfusion of MNPs in rats [22], we assembled the gypsum cubes within the FOV to construct a spatially 2D MNP distribution representing a biological distribution of a rat’s liver and heartThe distribution consisted of two distinct regions of MNP accumulation, divided as follows: 9 ($3{\mathrm{N}}_{\mathrm{x}}\times 3{\mathrm{N}}_{\mathrm{y}}$) gypsum cubes with a total nominal amount ${\mathrm{X}}_{\mathrm{M}\text{NP,nom}}$= 7.2 mg, and a single gypsum cube with ${\mathrm{X}}_{\text{MNP,nom}}$ = 4.8 mg, as shown in Fig. 7. It is worth mentioning thatwe exhibited the MNP 2D reconstruction through the experimental data collected. Besides, we set a phantom that did not fit the position of our reference samples in the voxel grid.

### Evaluation of quantitative imaging quality

We considered two parameters to assess the quality of the reconstructed quantitative images. The correlation coefficient *R* (Eq. 4) was employed to determine the geometric agreement between the nominal ($${X}_{MNP,nom}$$) and estimated MNP distributions ($${X}_{MNP,est})$$. Furthermore, we measured the accuracy of total quantification by the relative percentual difference $${X}_{diff}$$ between $${X}_{MNP,nom}$$ and $${X}_{MNP,est}$$, defined as $${X}_{MNP,diff}$$ (Eq. 5).

4 $$R=\frac{\sum_{k}\left({X}_{MNP\;nom,k}-\left({\overline{{\varvec{X}}} }_{{\varvec{M}}{\varvec{N}}{\varvec{P}},{\varvec{n}}{\varvec{o}}{\varvec{m}}}\right)\right).\left({X}_{est,k}-\left({\overline{{\varvec{X}}} }_{{\varvec{M}}{\varvec{N}}{\varvec{P}},{\varvec{e}}{\varvec{s}}{\varvec{t}}}\right)\right)}{\sqrt{\sum_{k}{\left({\widehat{X}}_{MNP\;nom,k}-{\overline{{\varvec{X}}} }_{{\varvec{M}}{\varvec{N}}{\varvec{P}},{\varvec{n}}{\varvec{o}}{\varvec{m}}}\right)}^{2}}.\sqrt{\sum_{k}{\left({X}_{est,k}-{\overline{{\varvec{X}}} }_{{\varvec{M}}{\varvec{N}}{\varvec{P}},{\varvec{e}}{\varvec{s}}{\varvec{t}}}\right)}^{2}}}$$

5 $${X}_{diff}=100\left(\frac{{\sum }_{k}{X}_{MNP,est}-{\sum }_{k}{X}_{MNP,nom}}{{\sum }_{k}{X}_{MNP,nom}}\right)$$

## Results

**Fig. 2:**
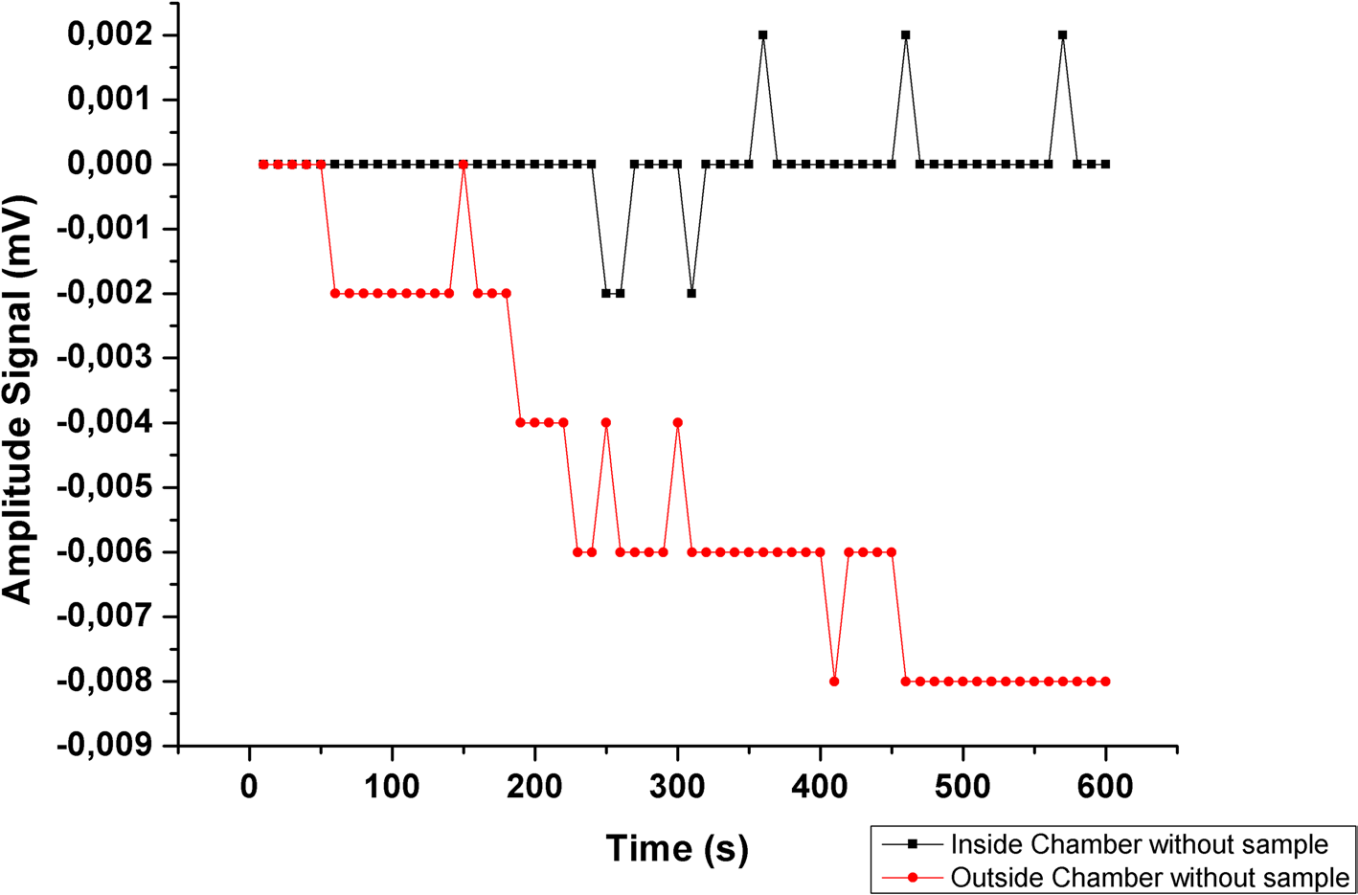
Stability test, showing the ACB signal intensity oscillating over time, inside (black dots) and outside (red dots) the shielding room

Figure 2 shows the oscillation in signal intensity over time, without any magnetic material near the pick-up coils, both outside and inside the shielding chamber.

As Fig. 2 indicates, the shielding room improved the stability of the central pick-up coil over time. The signal intensity oscillation was four times higher outside the shielded room for 10 min of acquisition. The instability sources associated with a non-shielded environment, such as electronic devices and Earth's magnetic field, showed no significant difference in intensity and orientation profiles around each pick-up coil. However, the signal amplitude registered in vivo experiments with 1 mL of magnetic nanoparticles at a concentration of 40 mg/mL is usually 100 times de noise level obtained. Thus, an assessment of the cost/benefit ratio must be considered, deciding between the level of noise and precision in the experiment and the system's simplicity*.*

**Fig. 3:**
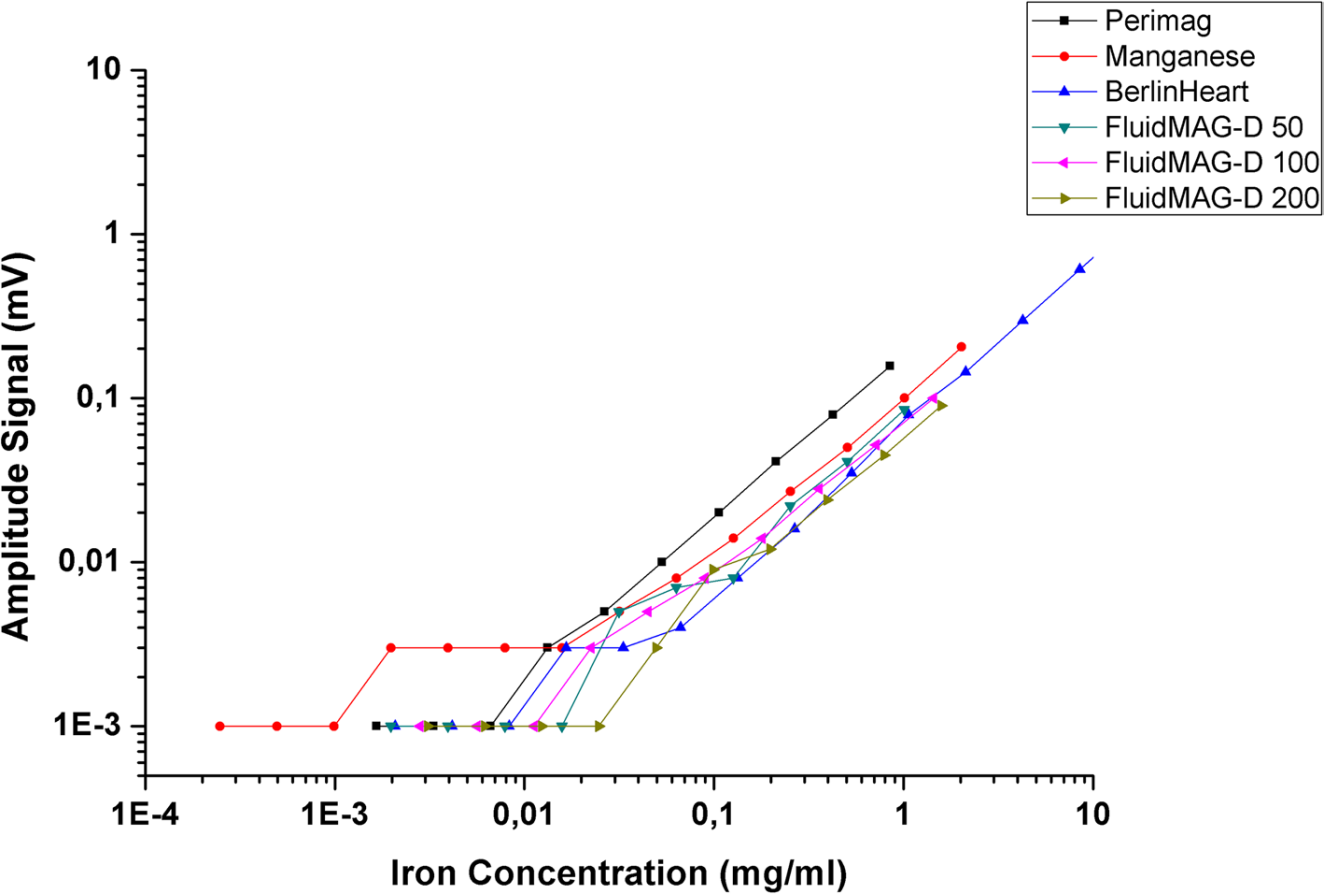
MC-ACB signal intensity from each MNPs conjugation with different concentrations

In our assess the ACB system's response to different conjugations of magnetic nanoparticles commercially available, we tested all samples distanced 5 mm from the sensor's surface. The initial concentration was 10 mg/mL (or less, depending on the availability of MNPs). Then, we diluted the sample until no signal was detected. Figure 3 shows the results obtained.

**Fig. 4:**
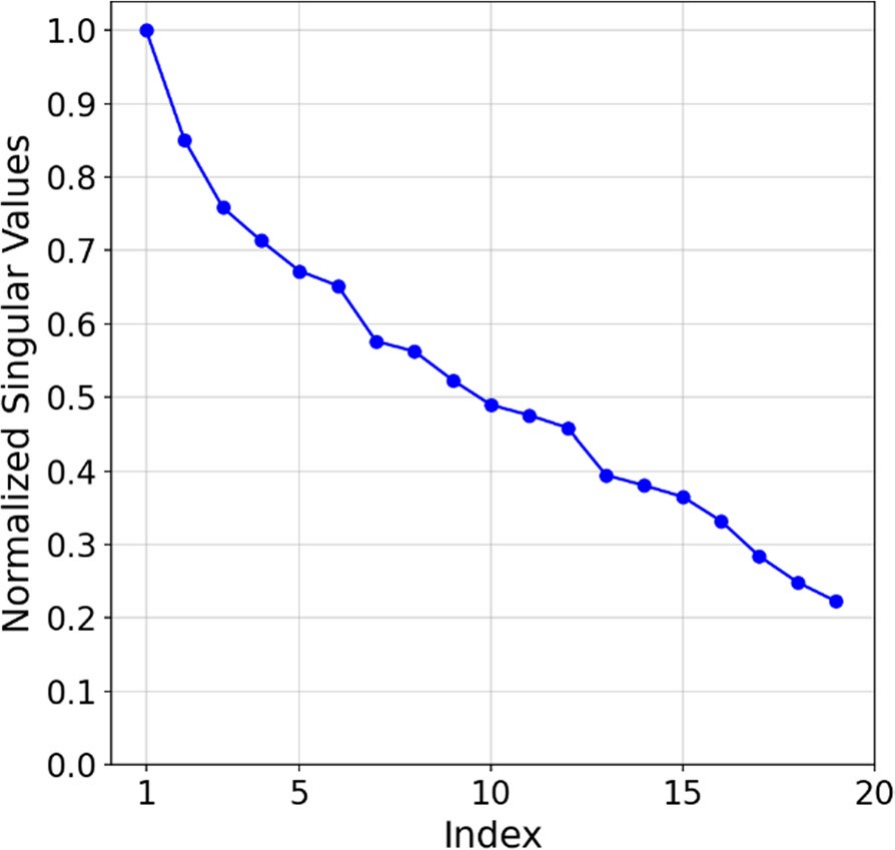
Singular values normalized to the largest singular value for multi-channel ACB systems with the truncation threshold used in TSVD for the model inversion

We determined the inverse solution's stability by the sensitivity matrices' singular values. The TSVD method calculates from the pseudo-inverse matrix ***L***^**+**^ the truncated singular values of the sensitivity matrix ***L*** as parameters. As a result, the maximum number of voxels containing MNPs, which can be simultaneously reconstructed, is determined by the number of non-zero singular values above a truncation threshold. Therefore, the obtained normalized singular values for the MC-ACB system using a voxel of $$1 \times 1\times 1.2$$ cm^3^ are shown in Fig. 4. The sensitivity matrix ***L*** for the multi-channel ACB system is of dimensions matrix $${\varvec{L}}\in {\mathbb{R}}^{196\times 19}$$ nd the optimal truncation threshold value (based on $${X}_{MNP,diff})$$ was found to be 20%, considered the threshold for system sensitivity. The fast decay is resulted from the system of equations, which provides only 19 equations to solve a FOV containing 196 voxels. This situation is referred to as an ill-conditioned problem.

Regarding the arrangement of the detection coils of the MC-ACB system (circular) and the rectangular FOV used, we simulated a sensitivity map of both proposed geometry and a squared array with 25 pick-up coils. It is worth pointing out that the coils simulation has the same length, number of turns, and electronic parameters in this simulation.

**Fig. 5:**
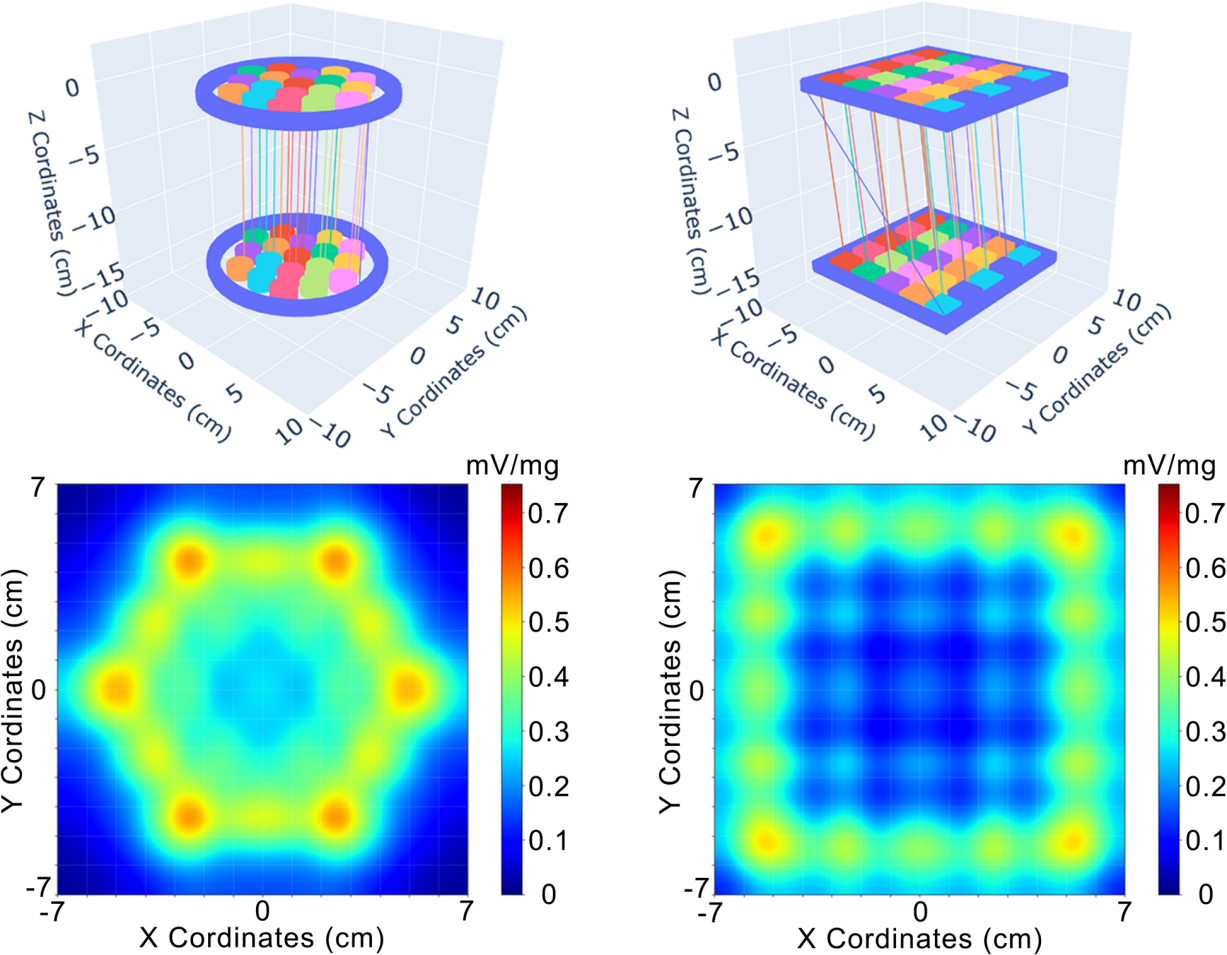
Spatial representations of the coordinates of each MC-ACB sensor (**A**) and simulation of a rectangular array of coils in a FOV of $14\times 14\times 1.2 c{m}^{3}$. Sensibility map of the circular (**C**) and the rectangular array of the coils (**D**). The scale was settled in $i=1 A$, $\omega =1 {s}^{-1}$ and ${\chi }_{m}=1kg/{m}^{3}$

Figure 5 shows the sensibility map of the proposed methodology. The sensor cannot assess magnetic materials along the corners due to the low sensitivity in these zones. In our setup arrangement of detection coils and FOV, the sensor cannot assess magnetic materials along the corners due to the low sensitivity in these zones.

Although rectangular geometries of coils can increase the effective area of detection and enable to detect in the corner of FOV, there are problems associated with less homogeneity along the FOV due to radial asymmetry. Some works showed that the spatial inhomogeneity around the FOV is associated with the worst inverse problem solutions in biomagnetic measurements. Furthermore, the exciting rectangular coil provides less sensitivity in the central area of FOV. We used circular geometries to avoid these problems. We emphasize that the method proposed in this work is a standard model for ACB real-time quantitative imaging, in which there is the possibility to optimize the electronic parameters, number, and geometries of the coils to enable different applications in future works.

The main advantage of using rectangular FOV is the high computational performance in reconstructing images, once the presence of non-responsive voxels in the reconstructed distribution does not show worst results when compared to a hexagonal FOV without these voxels.

**Fig. 6:**
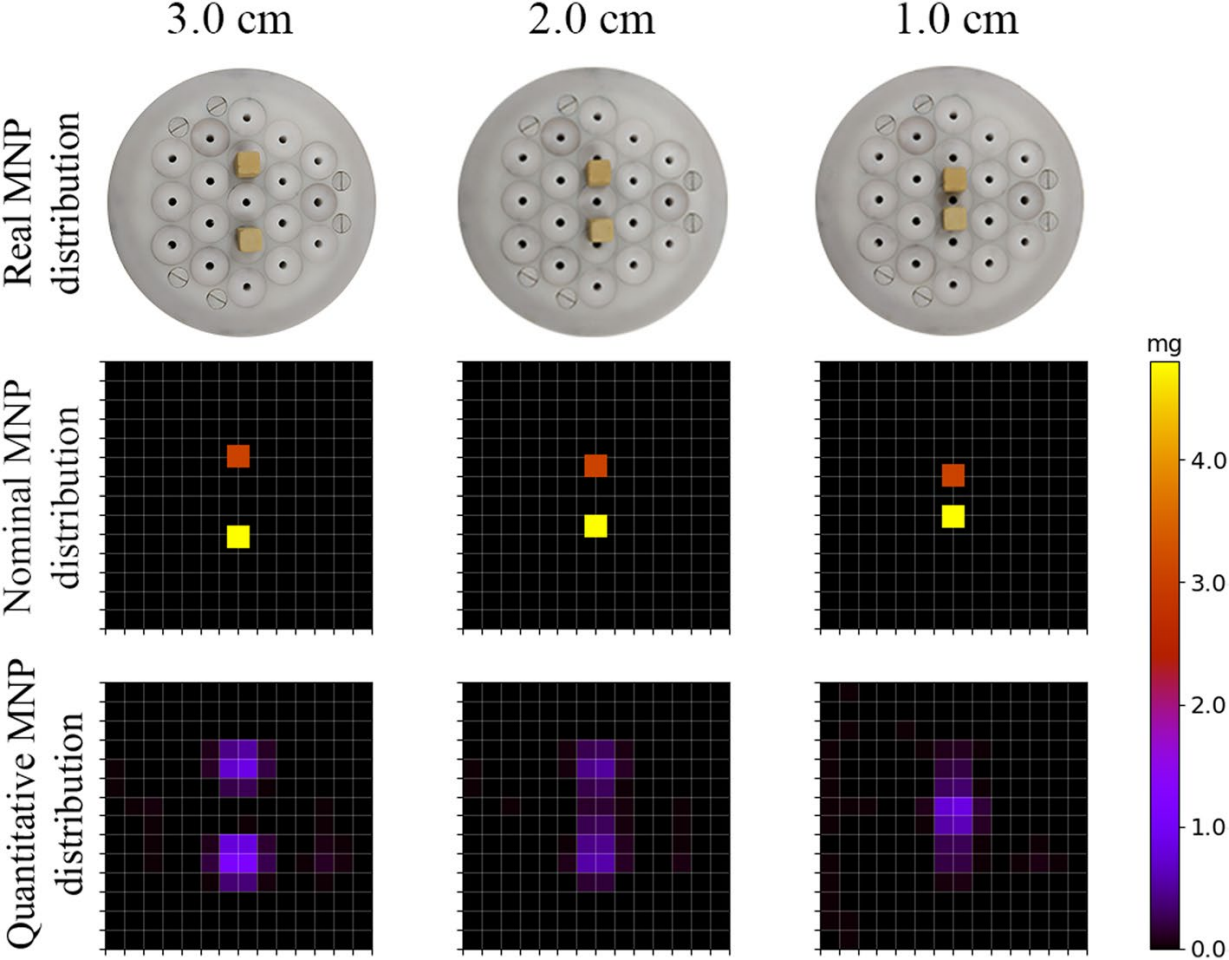
Nominal (top row) and reconstructed quantitative images (bottom row) of MNP cubes separated by 1, 2, and 3 cm (surface-to-surface distance)

To estimate the system's spatial resolution, we investigated how the ACB system resolves two cubes depending on their distance. Figure 6 shows the nominal distribution (top row) and the reconstructed MNPs distributions (bottom row) with two MNP-filled cubes separated by 1, 2, or 3 cm. The MC-ACB system could resolve the two cubes at a distance of 2 and 3 cm. However, for a distance of 1 cm, the reconstruction of the two cubes appears as a single and more concentrated source. For all three distances, the MNP distributions exhibited an overall correlation above 0.6 and a quantification difference ${\mathrm{X}}_{\mathrm{MNP},\mathrm{diff}}$ below 20% between the cubes, as indicated in Table 1. The better correlation result for a distance of 1 cm compared to a distance of 2 cm is mainly due to the higher homogeneity and sensitivity in the FOV on the cube's positions. Also, in the 1 cm measurement, the cubes are symmetric distributed along four voxels; in the 2 cm measurement, the cubes are asymmetrically distributed along four voxels. The asymmetric distribution in sub-voxel areas causes instability in inverse problem solution due to spatial resolution limitation.

**Table 1:** Correlation coefficient R and relative percentual differences${\mathrm{X}}_{\mathrm{MNP},\mathrm{diff}}$of the quantitative images for the two nominal MNP cubes separated by 1, 2, and 3 cm (surface-to-surface distance)

Distance (cm)	3.0	2.0	1.0
Correlation (R)	0.88	0.6	0.72
${X}_{MNP,diff}$(%)	13	13.1	16.8

**Fig. 7:**
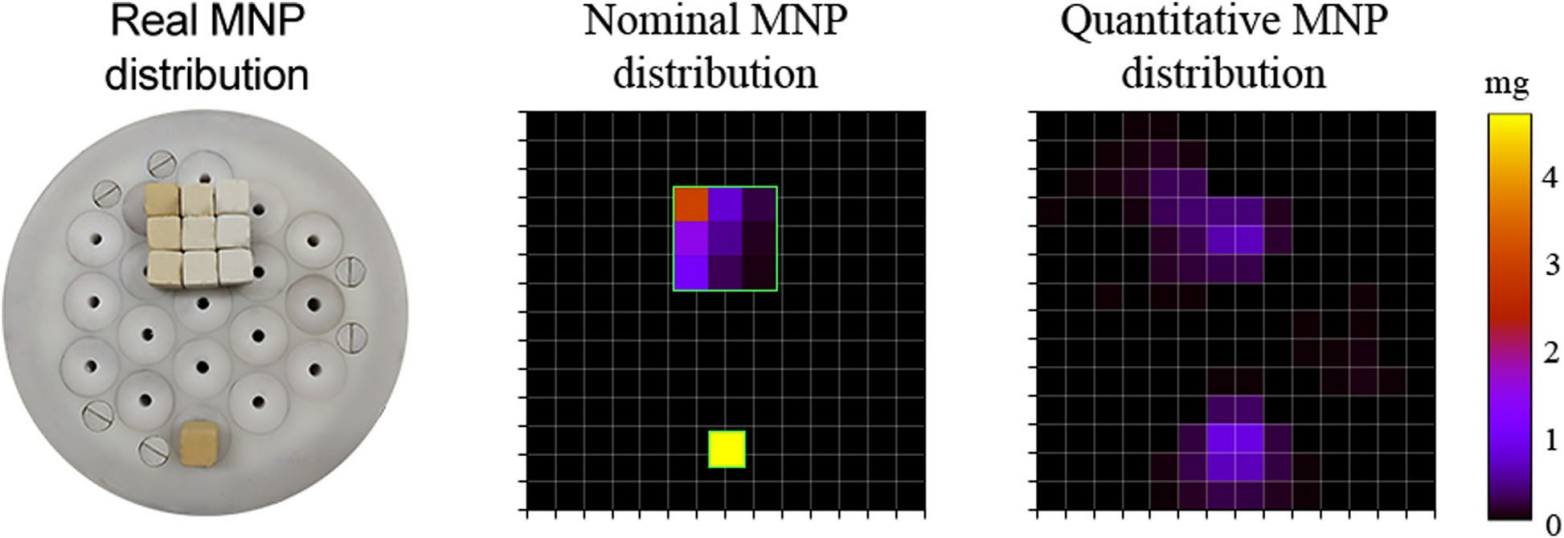
Nominal (left) and reconstructed quantitative image (right) of two different MNP distributions composed of gypsum cubes containing different amounts of MNP. For better visibility, the regions containing the cubes are highlighted in green

The quantitative reconstruction of the assembled 2D MNP distribution is presented in Fig. 7. We obtained a correlation of 0.77, which was satisfactory since our reconstructed MNP distribution is based on measurement data. Furthermore, the experimental phantom was arranged to not exactly match the cube positions previously established with sensitive matrix **L**, in which the voxel's position were shifted in the direction out of center cubes. However, even with the MNP distribution partially occupying some voxels, we found a ${X}_{MNP,diff}$ of 11%, below the values found in the spatial resolution test.

## Discussion

In a first step, we introduced MC-ACB to map the MNPs biodistribution without gaining any quantitative information [22]. Here, we extended the methodology utilizing the linear magnetization of MNP to enable quantitative MNPs imaging. Furthermore, as the real potential of the ACB system is the in vivo applications of MNPs in animals, we focused on developing the system envisaging the reconstruction of spatial MNPs distributions in rats. Therefore, we increased the number of pick-up coils with higher sensitivity and thereby the FOV of the system, which would ensure the system's capacity for in vivo applications. The MC-ACB enables the real-time quantitative imaging and assessment of MNP distributions to cover a rat in its x–y extension due to FOV of $14\times 14\times 1.2$ cm^3^ subdivided into 196 voxels of $1 \times 1\times$ 1.2cm^3^ with a reasonable spatial resolution of about 2 cm.

Through the MC-ACB, it is possible to reconstruct a more significant number of voxels, making the system an interesting tool in animal experiments since another magnetic system can reconstruct a limited number of voxels [10]. Even though the number of voxels used considerably exceeds the number of equations to solve the inverse problem, the values of the quality parameters indicated a strong correlation between the nominal and the estimated MNPs distributions and high image reconstruction quality. Moreover, for further applications, either in vitro or ex vivo measurements, the MC-ACB system could be adapted to a scanning approach, which would benefit from the number of multiple pick-up coils and the possibility of real-time acquisition. Regarding spatial resolution, improvements in the pick-up coil's diameter would lead to the current MC ACB setup being comparable to the ACB single-channel system as another magnetic measurement system. Moreover, the demand for magnetically shielded room implementation and the high system operation costs make the other magnetic systems, such as MPI and the MRX,expensive and less available to clinical environments.

On the other hand, the MC-ACB system can be implemented in any environment without the extra cost of shielding and maintenance. We also recognize that the first introduced ACB quantitative reconstruction of MNPs distributions employing a single-channel system yielded a more stable inverse problem due to the increased number of equations in the sensitivity matrix and reconstructed the MNPs distributions with higher spatial resolution due to the sensor’s dimensions. However, the scanning time process of approximately 10 min required by this setup limits the methodology to perform real-time measurements of a rat, while the multi-channel works in real-time at a 20 Hz sampling rate, enabling longer measurement times. Also, future improvements regarding the instrumentation and electronics as the lock-in amplifiers may increase the system portability and even reduce the costs, facilitating the use of the system in any environment. Although the MC-ACB presents high temporal resolution and an increased FOV, the current sensitivity limitation influences the stability of the inverse problem, reducing its performance in reconstructions and quantification. In this context, we decided to use the MNPs concentration at the mg/mL level to ensure that the ACB would have enough sensitivity to detect the samples. Besides, the literature reports showed in vivo studies involving the ACB in which the MNPs concentration ranged from 10 to 70 mg/ml [12, 14, 22, 23, 28]. Thus, the acquisition and reconstruction models proposed in this work are sensitive to in vivo MNP assays in rats. Furthermore, we recognize the high level of iron sample concentration used here once several studies suggested that MNPs at a concentration greater than 100 μg/mL are toxic to cells [29, 30]. We are currently developing some investigations based on other ACB arrangements to improve the system sensitivity in order to apply lower iron concentrations.

Despite the advantages of the methodology proposed, the current state of the art of MC-ACB presents considerable technical limitations compared to other quantitative MNP imaging modalities. MRX and MPI apply distinct spatial encoding strategies (*e.g.*, multiple excitations coils, magnetic field gradients, and frequency decode) to improve the inverse problem’s stability [31, 32]. To extend the ACB system to a tomography technique, similarly to MRX and MPI [6, 33], it is necessary to acquire additional information, mainly by adding coils out-of-detection plane. Therefore, reconstructing three-dimensional imaging can considerably enhance the quantitative reconstruction quality [6, 33]. A scanning approach [34] and multiple pick-up coils were the only strategies used in the ACB systems. Consequently, the lack of a more complex spatial encoding also restricts MC-ACB to 2D reconstructions. However, by evaluating the MNP pattern of accumulation and biodistribution of MNP, detailed biological investigations in vivo can be performed by ACB 2D quantitative reconstructions.

This work described a significant advance regarding the ACB methodology, which will enable further application studies involving MNPs applications and biomedical engineering and in pharmaceutics, gastroenterology, and physiology.

## Conclusion

In this study, we presented an MC-ACB system with nineteen pick-up coils and its employment to perform quantitative imaging of MNP distributions in a $14\times 14\times 1.2 {cm}^{3}$ FOV. Moreover, the system experimentally demonstrated the reconstruction of MNP content in two centimeters with sensitivity on the milligram scale. Despite the current limitations, the system proposed in this work does not require magnetic shielding compared to other systems.

Our group has extensive knowledge in drug delivery studies and gastroenterology assessments in animals and humans using the ACB methodology, focusing on assessing solid dosage forms in the through images [17, 35–38]. Our group also has studied the effects of pathologies, surgeries, and disturbed physiological states of the gastrointestinal transit in rats through images [39–43]. So far, signals of MNP accumulations in animal studies were only obtained by single sensor magnetic field monitoring, without image reconstruction. Even though we recently evaluated the gastrointestinal transit in animals through qualitative images, the proposed methodology was limited only to ex vivo measurements [34].

Furthermore, the MC-ACB system is a promising tool for quantitative imaging of MNP distributions in real-time, offering an affordable setup for easy access in clinical or laboratory environments.

## Data Availability

Almost all data are presented within the manuscript (figures and tables). The raw data presented in this study are available on request from the corresponding author.
